# The impact of the first wave of COVID-19 on students’ attainment, analysed by IRT modelling method

**DOI:** 10.1057/s41599-023-01613-1

**Published:** 2023-03-25

**Authors:** Rita Takács, Szabolcs Takács, Judit T. Kárász, Attila Oláh, Zoltán Horváth

**Affiliations:** 1 Faculty of Informatics, ELTE Eötvös Loránd University, Budapest, Hungary; 2 Department of General Psychology and Methodology, Institute of Psychology, Budapest, Hungary; 3 Károli Gáspár University of the Reformed Church in Hungary, Budapest, Hungary; 4 Doctoral School of Education, ELTE Eötvös Loránd University, Budapest, Hungary; 5 Institute of Education, ELTE Eötvös Loránd University, Budapest, Hungary; 6 Doctoral School of Psychology, ELTE Eötvös Loránd University, Budapest, Hungary; 7 Institute of Psychology, ELTE Eötvös Loránd University, Budapest, Hungary

**Keywords:** Psychology, Education

## Abstract

Universities around the world were closed for several months to slow down the spread of the COVID-19 pandemic. During this crisis, a tremendous amount of effort was made to use online education to support the teaching and learning process. The COVID-19 pandemic gave us a profound insight into how online education can radically affect students and how students adapt to new challenges. The question is how switching to online education affected dropout? This study shows the results of a research project clarifying the impact of the transition to online courses on dropouts. The data analysed are from a large public university in Europe where online education was introduced in March 2020. This study compares the academic progress of students newly enroled in 2018 and 2019 using IRT modelling. The results show that (1) this period did not contribute significantly to the increase in dropout, and we managed to retain our students.(2) Subjects became more achievable during online education, and students with less ability were also able to pass their exams. (3) Students who participated in online education reported lower average grade points than those who participated in on-campus education. Consequently, on-campus students could win better scholarships because of better grades than students who participated in online education. Analysing students’ results could help (1) resolve management issues regarding scholarship problems and (2) administrators develop programmes to increase retention in online education.

## Introduction

During the spread of the COVID-19 pandemic, several countries closed their university buildings and switched to online education. Some opinions suggest that online education had a negative effect on dropouts because of several factors, e.g., lack of social connections, poor contact with teachers. In bachelor’s programmes—like university courses in computer science—where dropout rates were high prior to the pandemic, many questions were raised about the impact of the transition to online education.

This study focuses on the effects of the first wave of the COVID-19 pandemic on students’ dropouts and performance in Hungary. Although the manuscript addresses academic dropout, other issues such as inequality or accessibility were also covered in the research.

### Theoretical background

#### Educational theory about student dropout in higher education

Tinto (1975) was the first researcher who analysed the dropout phenomenon and invented the interactional theory of student persistence in higher education. He (2012) highlighted the interactions between the student and the institution regarding how well they fit in academically and socially. Interactional theories suggest that students’ personal characteristics, traits, experience, and commitment can have an effect on students’ persistence (Pascarella and Terenzini, 1983; Terenzini and Reason, 2005; Reason, 2009). Braxton and Hirschy (2004) also emphasized the need for community on campus as a help of social integration to develop relationships between peers because interactions with other students and faculty members crucially determine whether students persist and continue their studies or leave.

The student dropout rate has been a crucial issue in higher education in the last two decades. Attrition has serious consequences on the individual (e.g., Nagrecha et al., 2017) at both economic (Di Pietro, 2006; Belloc et al., 2011) and educational (Cabrera et al., 2006) levels. As a worldwide phenomenon, it draws the attention of policy-makers, stake-holders and academics to the necessity of seeking solutions. The dropout crisis requires complex intervention programmes for encouraging students in order to complete their studies. Addressing such a dropout crisis requires an actionable interdisciplinary movement based on partnerships among stake-holders and academics.

According to Vision 2030 studies published by the European Union, education is vital for economic development because it has a direct influence on entrepreneurship and productivity growth; at the same time, it increases employment opportunities and women empowerment. Education helps to reduce unemployment and enhance students’ abilities and skills that will be needed in the labour market. Due to students’ high attrition, the economy also suffers because experts with a degree usually contribute more to the GDP than people without (Whittle and Rampton, 2020).

A comparative analysis of past studies has been conducted in order to identify various causes of students’ dropout. Students’ performance after the first academic year is a topic of significant interest: the lack of students' engagement in academic life and their unpreparedness are mainly responsible for dropout after the first highly crucial period. However, further studies are necessary to better understand this phenomenon.

### The characteristics of online education and its effect on dropout

Online education had already existed before the COVID-19 pandemic and had had a vast literature because online courses had been playing an important role in higher education. Online education has its own benefits, e.g., it enables students to work from the comfort of their homes with more convenient, accessible materials. In recent years, numerous investigations have been performed on how to increase the motivation of students by making them feel engaged during the learning processes (Molins-Ruano et al., 2014; Jovanovic et al., 2019). The other benefit is “humanizing”, which is an academic strategy that looks for solutions to improve equity gaps by recognizing the fact that learning situations are not the same for everyone. The aim of humanizing education is to remove the affective and cognitive barriers which appear during online learning and to provide a technique in higher education towards a more equitable future in which the success of all students is supported (Pacansky-Brock and Vincent-Layton, 2020). Humanizing online STEM courses has specific significance because creating such academic pathways can especially help the graduation of vulnerable, for example, non-traditional students. The definition of a non-traditional student belongs to Bean and Metzner (1985), who distinguished students by different characteristics. Non-traditional students are not on-campus students (but they can participate in online education), who are usually aged 24 years or older, and dominantly have a job and/or a family. Non-traditional students have less interaction with other participants in education, and they are much more influenced by other factors, e.g., family or other external responsibilities. Financial factors, family attitudes and external incentives can also influence dropout. The dropout model for non-traditional university students highlights that underperforming students are likely to leave the institution. Carr (2000) (in Rovai, 2003) noticed that persistence in online courses is regularly 10–20% lower than in on-campus courses. The dropout rate differs from institution to institution: some reports claim that 80% of students graduated, whereas other findings show that less than 50% of students completed their courses. Humanizing recognizes that engagement and accomplishment are the key factors in students’ success. Engagement and achievement are social constructs created through students’ experience. Teachers can help students to socialize and adapt to the academic environment by using humanizing practices like a liquid syllabus. Stommel (2013) also considers that hybrid pedagogy is a useful tool in order to support students’ learning because it helps teachers to implement new learning activities and facilitate collaboration among students.

Despite the various benefits that online education has, the success of students depends on the student’s capacity to independently and effectively engage in the learning process (Wang et al., 2013). Online learners are required to be more autonomous, as the exceptional nature of online settings relies on self-directed learning (Serdyukov and Hill, 2013). It is therefore especially critical that online learners, compared to their conventional classroom peers, have the self-generated capacity to control and manage their learning activities.

Online education also needs extra attention because the dropout rate is high in online university programmes. Students in online courses are more likely to drop out (Patterson and McFadden, 2009; in Nistor and Neubauer, 2010). Numerous studies reported much higher dropout rates than in the case of on-campus courses (Willging and Johnson, 2019; Levy, 2007; Morris et al., 2005; Patterson and McFadden, 2009; in Nistor and Neubauer, 2010). Many factors that lead to dropout were examined in the past. During online courses, students are less likely to form communities or study groups and the lack of learning support can lead to isolation. Consequently, demotivated students who were dedicated to their chosen major, in the beginning, may decide to drop out. Fortunately, there are different ways to support students who study in an online setting depending on their various psychological attributes. These psychological attributes that are connected to dropout have already been examined. One of the most noticeable hypothetical models of university persistence in online education was proposed by Rovai (2003). He claims that dropout depends on students’ characteristics e.g., learning style, socioeconomic status, studying skills, etc. Besides these factors, the method of education also has an impact on students’ decisions on whether they complete the course or drop out.

It is vital to distinguish the online education that was introduced as a consequence of the COVID-19 lockdown, when universities were forced to move their education to fully online platforms because online education had already existed in some educational institutions.

### The COVID-19 pandemic and its effect on education: Inequalities in home learning and colleges’ provision of distance teaching during school closure of the COVID-19 lockdown

The lives of millions of college students were affected not only by the health and economic implications of the COVID-19 pandemic but also by the closure of educational institutions. Home and academic environments were interlaced, and most institutions were caught unprepared. In this article, we examine the effects of the transition to online learning in areas such as academic attainment.

There are several debates on the effectiveness of moving to online education. Since currently there is little literature about the COVID-19 pandemic in relation to how it affects dropouts at universities, it is worth discussing it in order to have an overview of recent studies on students’ performance. The learning environment changed radically during the first wave of the pandemic in the spring semester of 2020. The transition to home learning and teaching in such a short time without any warning or preparation raised concerns and became the focus of attention for researchers, teachers, policymakers, and all those interested in the educational welfare of students.

A potential learning loss was anticipated, possibly affecting students’ cognitive gains in the long term (Andrew et al., 2020; Bayrakdar and Guveli, 2020; Brown et al., 2020); in fact, an increasing number of studies suggested that the lockdown might have far-reaching academic consequences (Bol, 2020). In general, results suggest that students’ motivation was substantially affected by the COVID-19 pandemic and that academic and relational changes were the most notable sources of stress on both the students’ side (e.g., Rahiem, 2021) and the teachers’ side (e.g., Abilleira et al., 2021; Daumiller et al., 2021). Engzell et al. (2021) examined nearly 350,000 students’ academic performance before and after the first wave of the pandemic in the Netherlands. Their results suggest that students made very little development while learning from home. Closures also had a substantial effect on students’ sense of belonging and self-efficacy. Academic knowledge loss could be even more severe in countries with less advanced infrastructure or a longer period of college closures (OECD, 2020).

Many researchers started to examine the effects of the COVID-19 pandemic on university students’ mental health and academic performance. Clark et al. (2021) claim that university students are increasingly considered a vulnerable population, as they experience extremely high levels of stress. They draw attention to the fact that students might suffer more from learning difficulties. Daniels et al. (2021) used a single survey to collect retrospective self-report data from Canadian undergraduate students (*n* = 98) about their motivation, engagement and perceptions of success and cheating before COVID-19, which shows that students’ achievements, goals, engagement and perception of success all significantly decreased, while their perception of cheating increased (Daniels et al., 2021). Other studies claim that during the COVID-19 pandemic, students were more engaged in studying and had higher perceptions of success. Studies also show that teachers’ strategies changed as well because of the lack of interaction between teachers and students, which led to the fact that students experienced more stress and were more likely to have difficulties in following the material presented and it could be one of the reasons for poor academic performance. Mendoza et al. (2021) investigated the relationships between anxiety and students’ performance during the first wave of the pandemic among college students. Anxiety regarding learning mathematics was measured among mathematics students studying at the Universidad Nacional de Chimborazo (UNACH) during the autumn semester of the academic year 2020. The total sample contained 120 students, who were studying the subject of mathematics at different levels. The results showed that there were statistically significant differences in the understanding of the contents presented by the teachers in a virtual way. During the COVID-19 pandemic the levels of mathematical anxiety increased. Teaching mathematics at university in an online format requires good quality digital connection and time-limited submission of assignments. This study draws attention to the negative result of the pandemic, i.e. the levels of anxiety might be greater during online education and not only in mathematics education but also in other subjects. Thus it could have an effect on students’ academic performance. However, the results are contradictory to what Said (2021) found, i.e. there was no difference in students’ performance before and during the COVID-19 pandemic. In their empirical study, they investigated the effect of the shift from face-to-face to online distance learning at one of the universities in Egypt. They compared the grades of 376 business students who participated in a face-to-face course in spring 2019 and those of 372 students who participated in the same course fully online in spring 2020 during the lockdown. A *T*-test was conducted to compare the grades of quizzes, coursework, and final exams of the two groups. The results suggested that there was no statistically significant difference. Another interesting result was that in some cases students had a better performance during the COVID-19 pandemic. At a large public university in Spain, Iglesias-Pradas et al. (2021) analysed the following instruction-related variables: class size, synchronous/asynchronous delivery of classes, and the use of digital supporting technologies on students’ academic performance. The research compared the academic results of the students during the COVID-19 pandemic with those of previous years. Using quantitative data from academic records across all (*n* = 43) courses of a bachelor’s degree programme, the study showed an increase in students’ academic performance during the sudden shift to online education. Gonzalez et al. (2020) had similar results. Their research group analysed the effects of COVID-19 on the autonomous learning performance of students. 458 students participated in their studies. In the control group, students started their studies in 2017 and 2018, while in the experimental group, students started in 2019. The results showed that there was a significant positive effect of the COVID-19 lockdown on students’ performance: students had changed their learning strategies and improved their efficiency by studying more continuously. Yu et al. (2021) found similar results. They used administrative data from students’ grade tracking systems and found that the causal effects of online education on students’ exam performance were positive in a Chinese middle school. Taking a difference-in-differences approach, they found that receiving online education during the COVID-19 lockdown improved students’ academic results by 0.22 of a standard deviation (Yu et al., 2021).

Currently, there is little literature about COVID-19 in relation to how it affects students’ performance at universities, so it is worth discussing this aspect as well.

### Teachers’ approach to their grading strategies and shift to online education during the COVID-19 lockdown

There is a vast literature on the limits of the capacities and challenges of online education (Davis et al., 2019; Dumford and Miller, 2018; Palvia et al., 2018). The lockdown during the COVID-19 pandemic created new challenges for teachers all over the world and called for innovative teaching techniques (Adedoyin and Soykan, 2020; Gamage et al., 2020; Paudel, 2020; Peimani and Kamalipour, 2021; Rapanta et al., 2020; Watermeyer et al., 2021). These changes had undoubtedly profound impacts on the academic discourse and everyday practices of teaching. Teachers’ motivations for maintaining effective online teaching during the lockdown were diverse and complex, and therefore, learning outcomes were difficult to be guaranteed. Yu et al. (2021) examined how innovative teaching could be continued during the COVID-19 pandemic, particularly by learning domain-specific knowledge and skills. The results confirmed that during the lockdown teachers who had studied online teaching methods improved their teaching skills and ICT (information and communication technology) efficacy.

Burgess and Sievertsen (2020) claim that due to the COVID-19 lockdown, educational institutions might cause major interruptions in students’ learning process. Disruption appeared not only in elaborating new knowledge but also in assessment. Given the proof of the significance of exams and tests for learning, educators had to consider postponing rather than renounce assessments. Akar and Coskun (2020) found that innovative teaching had a slight but positive relationship with creativity. From their point of view, it was not necessarily a consequence of shifting offline teaching to online platforms. Innovative teaching and digital technology were not granted and their impact on student’s performance or teachers’ grading practices is still unclear. The present research aimed to analyse *students’ attainment* during the COVID-19 pandemic by using student performance data. We focused on the relationship between participation in online courses and dropout decisions, which is connected to teachers’ grading. Examining how grades changed during the lockdown could give us an interesting insight into the educational inequality caused by online education regarding the scholarship system based on student’s grades.

### Research questions

We know very little about the effects of transitioning to online education on student dropout and teachers’ grading practices. Even less information is available on the relationship between COVID-19 and dropout, so it is worth a discussion due to the existing controversial and interesting studies on students’ performance. This article gives a suggestion on how the scholarship system could be changed and how we could avoid inequality caused by online education. There is a scholarship system in Hungary that provides financial support to full-time programme students, based on their academic achievement.

Another issue we discuss in this article is dropping out from university programmes, which is a crucial issue worldwide. Between 2010 and 2016 at a large public university in Europe (over 30,000 students) the overall attrition rate is 30%, with the Faculty of Informatics having the worst results (60%) but nowadays these figures are more promising (30|40%). These days at least 800,000 computer scientists may be needed in Europe (Europa.eu, 2015), but it seems to be a worldwide issue (Borzovs et al., 2015; Ohland et al., 2008) to retain students.

This study focuses on the effects of the first wave of the COVID-19 pandemic on students’ dropout and performance in Hungary. Although the manuscript addresses academic dropout, other issues such as inequality or accessibility are also covered in the research. The aim of the paper is therefore to investigate the following questions:

It is inconclusive whether the COVID-19 pandemic had negative effects on students’ performance, which is why we claim that

Hypothesis 1: There is a significant difference in grade point averages between students who participated in online education and those in on-campus education in the second semester of their studies.

Academic achievement (in both traditional and online learning settings) can be measured by accomplishing a specific result in an online assignment and is commonly expressed in terms of a grade point average (GPA; Lounsbury et al., 2005; Richardson et al., 2012; Wang, 2010). According to meta-analyses, GPA is one of the best predictors of dropout (Richardson et al., 2012; Broadbent and Poon, 2015).

Hypothesis 2: In some subjects (Basic Mathematics practice, Programming, Imperative Programming lecture + practice, Functional Programming, Object-oriented Programming practice + lecture, Algorithms and Data Structures lecture + practice, Discrete Mathematics practice and Analysis practice), it was easier to obtain a passing grade in online education.

Hypothesis 3: More of the students who participated in online education dropped out than those who received on-campus education.

## Methods

### Difficulty and differential analysis of subjects

In the examined higher education system, a BSc programme has six semesters and every subject is graded on a five-point scale, where 1 means fail, and grades from 2 to 5 mean pass, with 5 being the best grade. In the analysis only the final grades were counted in each subject. It is important to see that in order to achieve better grades (or obtain sufficient knowledge), a subject really needs differentiation. It is worth examining the subjects of the various courses because—although there are grades—there is some kind of expected knowledge or skill that the subject should measure. Students are expected to develop these competencies or at least reach an expected level by the end of the semester. To find out whether this kind of competency actually exists (and was developed during online education) and whether the subjects measure this kind of competency, Item Response Theory (IRT) analysis was used to examine the subjects included in the computer science BSc programme. The aim of IRT analysis modelling is to bring the difficulty of the subjects and the ability of the students to the same scale (GRM, Forero and Maydeu-Olivares, 2009; Rasch, 1960). We had already successfully applied a special IRT model in order to analyse the effects of a student retention programme. In order to prevent student dropout, in a large public university in Europe, a prevention and promotion programme was added to the bachelor’s programme and an education reform was also implemented. In most education systems students have to collect 30 credits per semester by successfully completing 8|10 subjects. We conducted an analysis using data science techniques and the most difficult subjects were identified. As a result, harder subjects were removed, and more introductory courses were built into the curriculum of the first year. A further action—as an intervention—was added to a computer science degree programme: all theoretical lectures became compulsory to attend. According to the results, the dropout level decreased by 28%. The most important benefit of the education reform was that most subjects had become accomplishable (Takács et al., 2021).^1^

Hypothesis 1 claims that the online transition due to COVID-19 during the second semester of the 2019 academic year did not result in a change in the requirement system of the subjects. Hypothesis 2 claims that essentially the same expectations were formulated by teachers. In contrast, the way teachers evaluate students necessarily changed. A subject with a given difficulty could be passed by a student with the same ability level with a given probability. Obviously, all subjects that had been less difficult were more likely to be correctly passed than more difficult subjects. The analysis was performed using the IRT, based on the STATA15 software package.

In the study, 862 students were involved in the bachelor’s computer science programme. There were 438 (415) students who started on-campus education in 2018 and 447 students who started on-campus education in 2019, but from March 2020 they participated in online education (Table 1). Table 1 shows the result of Hypothesis 1: The grade point average of students who participated in online education (2.5) was lower than that of students who participated in on-campus education (3.3). Table 1 also shows that 447 students participated in online education and only 19 dropped out; 438 students started on-campus education and 50 dropped out. We can conclude that there was no significant difference between students’ dropping out who participated in online education and those who received on-campus education (Hypothesis 3). Note: We can conclude that the grade point average of students who participated in online education (2.5) was lower than that of students who participated in on-campus education (3.3) (Hypothesis 1). On the other hand, there was no significant difference between the drop-out rate of students’ who participated in online education and that of those who received on-campus education (Hypothesis 3). These case numbers make it unnecessary to apply any statistical evidence because the result is obvious.

**Table 1:** Descriptive statistics of students.

	2018 academic year (on-campus education)	2019 academic year (online education at the spring semester)
Total number of students who started the education	438	447
Number of students dropped out	50	19
Grade point average of the second semester	3.3	2.5

The subjects were examined by fitting a 2-parameter IRT model to them (scale 1–5 with grades, assuming an ordinal model using the STATA15 programme). ‘Grades’ mean the final grade of the subjects. The STATA15.0 software package was used for the analysis, and the Graded Response Model version of the Ordered item models was chosen from the IRT procedures (GRM; Forero and Maydeu-Olivares, 2009).

During the procedure, we examined two parameters: the difficulty of the items and the slope. We took into account those subjects for which the subject matter of the subject remained the same over the years, or the exams did not change substantially (exam grade, according to the same assessment criteria). However, it is important to note that obviously, not the same students completed the assignments each year.

The study involved the following subjects (only professional subjects were considered):

- Mathematical Foundations
- Programming
- Computer Systems lecture+practice
- Imperative Programming
- Functional Programming
- Object-oriented Programming lecture + practice
- Algorithms and Data Structures I. lecture
- Algorithms and Data Structures I. practice
- Discrete Mathematics I. lecture
- Discrete Mathematics I. practice
- Analysis I. L
- Analysis I. P

## Results

### Examination of slope and difficulty coefficients

In this section, we examine Table 2. As a first step, it is crucial to understand the slope indices of the given objects in different years, whether they change from one year to another. Table 2 shows the result of Hypothesis 2: In most subjects (Basic Mathematics practice, Programming, Imperative Programming lecture + practice, Functional Programming, Object-oriented Programming practice+lecture, Algorithms and Data Structures lecture + practice, Discrete Mathematics practice, and Analysis practice), it was easier to obtain a passing grade in online education.

**Table 2:** IRT-model on subjects of the first two semesters of CS degree programme.

2018/2019	2019/2020	2018/2019	2019/2020
*Mathematical foundations*		*Computer systems L* + *P*	
≥1 | −2.92	≥1 | −2.49	≥1 | −3.6	≥1 | −3.26
≥2 | −1.70	≥2 | −1.84	≥2 | −2.55	≥2 | −2.47
≥3 | −0.563	≥3 | −0.688	≥3 | −2.10	≥3 | −2.17
≥4 | 0.092	≥4 | 0.093	≥4 | −1.1≥	4 | −1.08
=5 | 0.856	=5 | 0.792	=5 | −0.242	=5 | 0.149
*Programming*		*Functional programming*	
≥1 | −2.59	≥1 | −2.27	≥1 | −2.08	≥1 | −1.57
≥2 | −1.68	≥2 | −1.62	≥2 | −1.41	≥2 | −1.2
≥3 | −1.57	≥3 | −1.53	≥3 | −0.731	≥3 | −0.592
≥4 | −0.692	≥4 | −1.08	≥4 | −0.221	≥4 | −0.147
=5 | 0.387	=5 | −0.251	=5 | 0.30	=5 | 0.356
*Imperative programming*			
≥1 | −2.59	≥1 | −2.27		
≥2 | −1.68	≥2 | −1.62		
≥3 | −1.57	≥3 | −1.53		
≥4 | −0.692	≥4 | −1.08		
=5 | 0.387	=5 | −0.251		
*Object-oriented programming L* + *P*			
≥2 | −0.851	≥2 | −0.655		
≥3 | −0.722	≥3 | −0.595		
≥4 | −0.02	≥4 | −0.296		
=5 | 0.747	=5 | 0.35		
*Algorithms and data structures I. L*			
≥1 | −1.74	≥1 | −1.47		
≥2 | −1.47	≥2 | −1.40		
≥3 | 0.013	≥3 | −0.53		
≥4 | 0.898	≥4 | 0.320		
=5 | 1.54	=5 | 0.77		
*Algorithms and data structures I. P*			
≥1 | −2.40	≥1 | −1.52		
≥2 | −1.7	≥2 | −1.24		
≥3 | −0.741	≥3 | −0.813		
≥4 | 0.023	≥4 | −0.280		
=5 | 0.746	=5 | 0.321		
*Discrete Mathematics I. Pr*		*Discrete Mathematics I. L.*	
≥1 | −2.35	>=1 | −2.5	>=1 | −1.71	>=1 | −1.44
≥2 | −1.54	>=2 | −1.74	>=2 | −1.43	>=2 | −1.08
≥3 | −0.612	>=3 | −1.15	>=3 | −0.728	>=3 | −0.452
≥4 | 0.14	>=4 | −0.308	>=4 | 0.498	>=4 | 0.922
=5 | 0.853	=5 | 0.506	=5 | 1.17	=5 | 1.16
*Analysis I. P.*		*Analysis I. L.*	
≥1 | −1.65	≥1 | −1.57	≥1 | −1.6	≥1 | −1.8
≥2 | −1.27≥	2 | −1.29	≥2 | −1.48	≥2 | −1.68
≥3 | 0.093	≥3 | −0.573	≥3 | −0.230	≥3 | 0
>=4 | 0.709	≥4 | 0.055	≥4 | 0.572	≥4 | 1.89
=5 | 1.4	=5 | 0.920	=5 | 1.24	=5 | 2.34

Two parametric procedures were applied: each subject has a difficulty index and a slope.

**Fig. 1:**
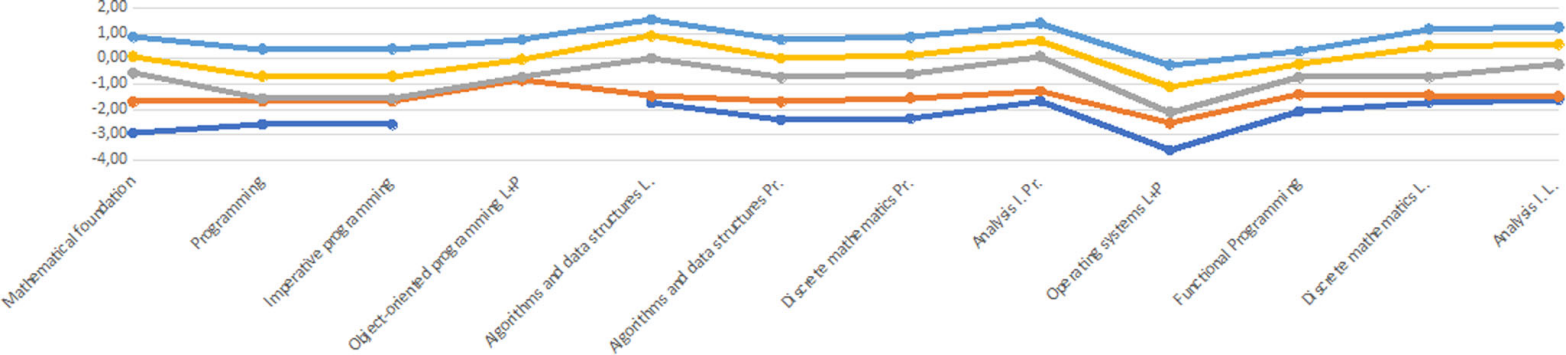
Difficulty levels of the subjects in 2018 and 2019 academic year.

While if the student’s ability falls short of the difficulty, the denominator of the fraction will increase, so the probability that the student will be able to pass the exam will increase—they will earn a good grade (Fig. 1).

Instead of introducing the whole subject network, we introduce a typical subject that was analysed using the IRT. The analyses of the subject of Discrete Mathematics enable us to adequately illustrate the classic phenomenon that arose. The complete analysis of the subjects can be found in Table 2.

The period before 2019 and after 2019 are shown separately in the table, as at the beginning of 2020 the lockdown took place when online education was introduced to all students so it had an impact on academic achievement. We presupposed that it had manifested itself in the subjects’ completing difficulty and in their ability to differentiate.

### Discrete mathematics I. practice

As far as the Discrete Mathematics subject is regarded, we can observe a slope of high value above 3 (sometimes 4) before and after 2019, which means that the subject had strong differentiating abilities both before and after the COVID-19 pandemic.

## Discussion

There is a debate in the literature on how the performance of students changed during online education. Whereas Said (2021) found no difference in students’ performance before and during the COVID-19 pandemic, the study by Iglesias-Pradas et al. (2021) showed an increase in students’ academic performance in distance education. Gonzalez et al. (2020) predicted better results during online education than in the case of on-campus education. This study partly confirmed their result because more students tried taking the exams. However, they could not perform better as predicted by Gonzalez et al. (2020) because among computer science students those who participated in online education obtained lower grade point averages than those who participated in on-campus education. According to our results, grade point averages differed substantially between the two examined groups (Hypothesis 1). It can be seen that there are no significant differences in the study groups in terms of dropout after the first year of studies, and the number of students affected was not substantially higher/lower. There are no significant differences in dropout rates between students participating in on-campus or online education (Hypothesis 3).

The result above is crucial; however, the implications and prospective steps based on this result are even more important.

It can be seen that with the introduction of online education, more teaching and learning strategies became available for certain subjects. Teachers’ grading strategies as well as their intentions when giving grades can be assumed as the possible reasons behind the grades. These strategies on both sides (teachers’ and students’) may have appeared during online education.

There were basically two types of changes regarding the grades for the different subjects:

1. The difficulty associated with the particular grade of the subject in online education decreased for each value on a scale of 1–5 for a given subject (Hypothesis 2). This means that even failing (grade 1) was easier (students preferred to try the exam even if they were unprepared), or even obtaining other passing grades was easier, too. It should be noted that the examined phenomenon cannot have a negative slope (typically not 0), because a slope of 0 means that there is ½ of a probability (regardless of ability) that a student passes a given exam. Fortunately, this is not the case, so we can assume that all slopes are positive.

- (a) Behind this strategy, in the case of grade 1, it can be assumed that in online education students’ general strategy was to register for the exam and try it even if unprepared in contrast to the on-campus student who would not take the exam if s/he was unprepared.
- (b) It seems that it became easier to obtain a passing grade. Behind this phenomenon, strategies can be assumed from both faculty members' and students’ sides. In case of failing the exam, it makes no sense to talk about the strategy of the teacher, because the teacher was more likely to give a passing grade or even a better grade for less knowledge. In general, the thresholds for obtaining the grade were lower in all cases. This could have been illustrated by the following subjects: Basic Mathematics practice, Programming, Imperative Programming lecture + practice, Functional Programming, Object-oriented Programming practice + lecture, Algorithms and Data Structures lecture + practice, Discrete Mathematics practice and Analysis practice.

2. Analysing further the subjects by IRT modelling, we saw that it was easier to obtain lower grades (grades 1, 2 and 3). However, in the case grade 4 or 5, it appears that it was more difficult to obtain them due to the prevalence of the higher requirements of the subjects.
  (a) The insufficient grades’ (i.e. grade 1) lower level of difficulty (shown by the IRT model) clearly showed that there was no substantial difference in this respect compared to obtaining insufficient grades during the on-campus or online education period.
  (b) The results showed that obtaining good grades (4 or 5) became more difficult during online education. It can be assumed that students participating in online education require some kind of help from education management in order to compensate for the disadvantages posed by distance learning because they got worse grades and worse average grade points as compared to on-campus students.
  In the following, we examine what strategies faculty members and students may apply considering the difficulty of each grade of the subjects (left column of Table 2) showed a decreasing trend.
  1. From the students’ point of view, isolation could result in students being involved in studying more effectively. Consequently, the time spent on the elaboration of the subjects may increase (Wang et al., 2013) compared to in-class education and by using available materials, textbooks, practice assignments, students could devote extra energy to subjects, which may result in better exam grades.
  2. From the teachers’ point of view, teachers might want to offer some ‘compensation’ at exams due to non-traditional teaching. In light of this, they are likely to ask a ‘slightly easier’ question, adapt them to the practice tasks, or even lower the exam requirements, e.g., lowering the score limits by 1-2 points more favourable, or accepting answers that would not be accepted in other circumstances.
  3. Note that these two strategies may have been present at the same time: the teacher perceived increased student contribution during the semester, for example, greater activity in online classes, and therefore, provided them with some reward by giving better final grades after taking into consideration their overall performance during the semester.

Please note that both narratives could appear at the same time.

It is also important to see that although grade point averages shifted, the shift was not necessarily drastic, and dropout rates did not improve. It may also be legitimate that there were individual characteristics that caused the difference in the grade point average.

1. From the student’s point of view, it could also mean that they were prepared in the same way in online education as in in-class education for exams. However, the same strategy did not necessarily result in better grades in the upper segment (obtaining 4 or 5).
2. The teacher determined the minimum level of requirements, either for mid-term achievements or final assignments and communicated it clearly to the students. How to obtain a passing grade was clear to the students. However, how to obtain good and excellent grades would have required more serious preparation and self-directed learning in online settings.

It is important to see that subjects, where it was more difficult to obtain better grades, were mainly theoretical ones (e.g.: lectures). They were tested mostly by oral exams where it was not possible to use additional materials, they had to answer directly to the questions. In this respect, teachers’ explanations, for example, could lead to very serious shortcomings in the case of knowledge transfer as well as the transfer of the same levels of the previous examination systems. This could result in lower achievement in areas where teachers’ explanations would have been necessary. Students had a harder time bridging the online-offline gap.

### Education management issues

In the higher education system analysed, students receive a scholarship according to their grade point average achievement. It is calculated based on the average of the final grades received at the end of the semester and the credits earned. It is worth considering that for online systems, credit-weighted averages will not necessarily show students’ real knowledge. This also results in serious problems when it comes to rewarding students’ performance with a scholarship, where multiple types of educational models may conflict.

This is because whether students can successfully complete a subject differs greatly in an online education system but subjects seem to have become fundamentally easier.

Thus, different education systems (in-class education and online education) can lead to different grading results, so it is not advisable to apply the same scholarship system because it can be fundamentally unfair (some fields can become easier or more difficult).

## Conclusion

The results of this study imply that COVID-19 had various effects on the education sector. The results are discussed in connection with the introduction of online education during the COVID-19 pandemic in terms of dropouts. The teachers who were involved in this study were the same during online education and on-campus education. This is the reason why we can conclude that the results also seem to suggest that teachers tried to compensate for the negative effects of the pandemic by bringing in pedagogical strategies aimed at ensuring that students could more easily obtain passing grades in examinations. Similarly, according to Mendoza et al. (2021), the failures of online education had a direct impact on student’s performance and learning.

This study found that students achieved better results during in-class education, which offers interesting implications for teaching practice. The results suggest that organizational support and flexible structures are needed in order to adapt teaching to the new circumstances set by the crisis. Higher education institutions should pay careful attention to developing students’ skills as well as to seeking ways to quickly respond to environmental changes while sustaining the delivery of high-quality education.

In the literature review, contradictory results were found for students’ performance during online education; therefore, this result contends previous literature and should be further explored.

A substantial difference in grade point averages can be found between the two examined groups. The first hypothesis was confirmed: students who participated in on-campus education obtained better grade point averages than students of online education. The teachers declared the minimum level of requirement and communicated it to the students quite clearly. It is a thought-provoking result that for online education, credit-weighted grade point averages would not necessarily show real knowledge well.

The second hypothesis was also proved because some subjects became easier to pass in online education, at least obtaining a passing grade. Online education facilitated students’ strategies e.g., creating an agenda of studying was essential to maintain effective and continuous learning.

The third hypothesis was not confirmed because significant differences in dropout rates were not found between the students who participated in online education and on-campus education. The dropout rate remained nearly unchanged between students who participated in online education (19 students dropped out), and students who participated in on-campus education (50 students dropped out). Introducing online education was effective or at least not harmful in terms of dropout because the dropout rate remained unchanged, compared to the previous year.

The results suggest that regarding dropout rates, there was no significant difference between online and on-campus education. The result suggests several assumptions: e.g.: the teachers had been more indulgent, as they also found it more difficult to communicate effectively during the COVID-19 period and were less able to apply with traditional methods. The process of knowledge transfer moved to online platforms and a different kind of interaction could be applied to rely on the online education system.

### Limitations of the study and future research

This study proposed research clarifying the impact of the transition to online courses on dropout. The results show that this period did not contribute significantly to the increase in dropouts. Subjects became more achievable during online education. Students who participated in online education reported lower average grade points than students who participated in on-campus education. Consequently, on-campus students could win better scholarships than students who participated in online education because of better grades.

Several other factors e.g., whether students have met in person in the past, could affect the dropout and grade point averages which were not taken into consideration in this research. In the future, it is recommended to measure students’ current level of knowledge, how much they can adapt to online education, and how they would react in the next similar crisis.

Even though this study presents interesting results, the authors believe that the conclusions derived from them should be interpreted carefully. It allows both researchers and teachers to develop further methods to examine students’ strategies in online education during the COVID-19 period. Future research should be extended with additional variables. Data analysis techniques should also be taken into consideration in order to evaluate the academic profile of students who dropped out in previous years. Limitations include that analysis does not entirely reflect the true engagement of students in the education system because only the first two semesters were examined.

The results of this study open new lines of similar research. It is hoped that other researchers will consider examining the potential impact of COVID-19 on educational planning and scholarship systems. The results of this study can further be validated by considering a wider study that would collect both quantitative and qualitative data to give a deeper understanding of the effects of this epidemic.

## Data Availability

The datasets generated during and/or analysed during the current study are available from the corresponding author on reasonable request.

## Footnotes

1 For a detailed explanation of the method see Takács et al. (2021).
